# Reduced Cortical Thickness in Mental Retardation

**DOI:** 10.1371/journal.pone.0029673

**Published:** 2011-12-27

**Authors:** Yuanchao Zhang, Yan Wu, Maohu Zhu, Chao Wang, Jiaojian Wang, Yun Zhang, Chunshui Yu, Tianzi Jiang

**Affiliations:** 1 Key Laboratory for NeuroInformation of Ministry of Education, School of Life Science and Technology, University of Electronic Science and Technology of China, Chengdu, People's Republic of China; 2 LIAMA Center for Computational Medicine, National Laboratory of Pattern Recognition, Institute of Automation, Chinese Academy of Sciences, Beijing, People's Republic of China; 3 Department of Radiology, Tianjin Medical University General Hospital, Tianjin, People's Republic of China; University of Jaén, Spain

## Abstract

Mental retardation is a developmental disorder associated with impaired cognitive functioning and deficits in adaptive behaviors. Many studies have addressed white matter abnormalities in patients with mental retardation, while the changes of the cerebral cortex have been studied to a lesser extent. Quantitative analysis of cortical integrity using cortical thickness measurement may provide new insights into the gray matter pathology. In this study, cortical thickness was compared between 13 patients with mental retardation and 26 demographically matched healthy controls. We found that patients with mental retardation had significantly reduced cortical thickness in multiple brain regions compared with healthy controls. These regions include the bilateral lingual gyrus, the bilateral fusiform gyrus, the bilateral parahippocampal gyrus, the bilateral temporal pole, the left inferior temporal gyrus, the right lateral orbitofrontal cortex and the right precentral gyrus. The observed cortical thickness reductions might be the anatomical substrates for the impaired cognitive functioning and deficits in adaptive behaviors in patients with mental retardation. Cortical thickness measurement might provide a sensitive prospective surrogate marker for clinical trials of neuroprotective medications.

## Introduction

Mental retardation, also called developmental delay or mental delay, is a developmental disorder characterized by sub-average cognitive functioning and deficits in adaptive behaviors. This disorder is generally taken as corresponding to an intelligence quotient (IQ) <70 and has a prevalence of about 2–3% in the general population[1]. The underlying cause of mental retardation often remains unclear despite extensive clinical examination and investigations. Based on in vivo magnetic resonance imaging techniques, many researches have been conducted to identify brain structural abnormalities in mental retardation patients. Most of these studies were performed qualitatively and focused on the white matter alterations in mental retardation patients[2], [3], [4], while quantitative analysis of changes of the cerebral cortex have been done to a lesser extent [5]. Quantitative studies are necessary to further investigate the brain structural abnormalities in patients with mental retardation. According to previous neuropathologic researches, mental retardation pathologies are associated with neuronal losses and spine dysgenesis in multiple cortical regions [6], [7], [8]. Therefore, quantitative maps of cortical integrity of the mental retardation patients may contribute in understanding the pathogenesis of the disorder.

By manual outlining of region of interest (ROI), a previous study documented significantly reduced volume in patients with mental retardation when compared with normal controls in bilateral prefrontal lobes and bilateral temporal lobes [9]. However, ROI-based methods need a priori to define the region of interest and limit the identification of changes elsewhere in the cerebral cortex [5]. Moreover, manual outlining of the ROIs is labor-intensive and might introduce user bias. Given the above consideration, Moorhead et al. employed an automated methodology, voxel-based morphometry (VBM) [10], to further investigate this issue [11], [12]. In their study, significant reductions of regional gray matter volume were observed in the occipital lobes, the frontal lobes, the right temporal lobe and the bilateral parietal lobe in mental retardation patients. Interpreting such results however is difficult, given that the smoothing step of VBM can involve structures that are in close spatial proximity but not closely anatomically connected [13]. In addition, interpretation of the differences obtained by VBM can be difficult since it does not measure the actual physical characteristic directly [13].

Recently, advanced image processing approaches have been developed to measure cortical thickness by calculating the distance between the gray matter and white matter surfaces across the entire cortical mantle. Indeed, cortical thickness is a more direct and biologically meaningful measurement, which could reflect the size, density and arrangement of cells [14]. In previous neuroimaging studies, cortical thickness has been used to investigate structural changes in the neurodevelopmental process in healthy subjects [15], [16] as well as pathological changes in several diseased populations[13], [17]. Relationships between cortical thickness and IQ have also been explored by many researchers to determine the neuroanatomical substrates of human intelligence [14], [18]. Therefore, in this study, cortical thickness measurement was adopted to investigate cortical abnormalities in mental retardation. We contrasted the cortical thickness between normal controls and patients with mental retardation using a surface-based generalized linear model (GLM) tool to map group contrasts on a vertex-by-vertex basis. Based on previous studies [9], [11], we hypothesized that patients with mental retardation would exhibit cortical thinning in multiple brain regions including the occipital lobe and the temporal lobe.

## Results

**Figure 1:**
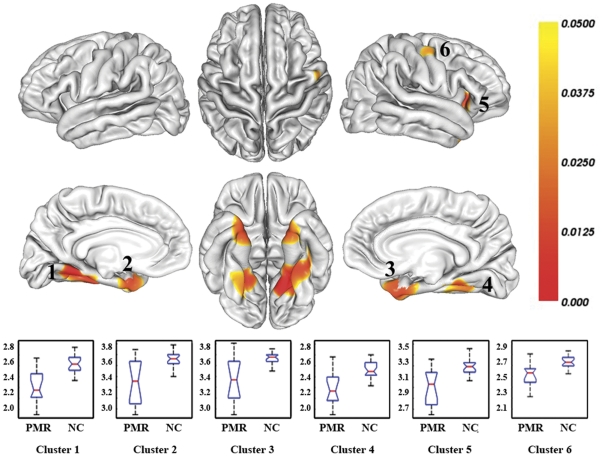
Brain regions with cortical thickness reductions in mental retardation and distribution of average cortical thickness (in mm) of each corresponding region. The results were corrected for multiple comparisons (P<0.05, the vertex-based RFT correction). The color bar indicates the corrected P-values. The integers are the cluster IDs corresponding to those of Table 1. Normal controls, NC; Patients with mental retardation, PMR.

Compared with normal controls, only reduced cortical thickness was observed in multiple brain regions in patients with mental retardation. We found six regions of difference with thresholds of P<0.05 (corrected) and cluster size > = 100 vertices. These regions included the bilateral lingual gyrus, the bilateral fusiform gyrus, the bilateral parahippocampal gyrus, the bilateral temporal pole, the left inferior temporal gyrus, the right lateral orbitofrontal cortex and the right precentral gyrus (Table 1). For visualization, regions of difference were projected onto the pial surface of the average template. (Figure 1)

**Table 1:** Regions with reduced cortical thickness in mental retardation.

Cluster ID	Anatomic regions	Side	Cluster size (vertices)	Peak P-value	MNI coordinates (x, y, z)
1	fusiform gyrus, parahippocampal gyrus, lingual gyrus, inferior temporal cortex	Left	3982	0.00036	−27.9, −49.1, −7.0
2	temporal pole	Left	1101	0.00842	−19.5, −0.7, −26.3
3	temporal pole	Right	1113	0.00940	23.1, 3.0, −40.4
4	fusiform gyrus, parahippocampal gyrus, lingual gyrus	Right	1815	0.00486	23.6, −46.3, −11.3
5	lateral orbitofrontal cortex	Right	850	0.00419	31.3, 24.7, −5.2
6	precentral gyrus	Right	280	0.02714	56.7, −1.4, 44.8

## Discussion

In this study, we used a surface-based approach to explore the differences in the cortical thickness between patients with mental retardation and demographically matched healthy controls. Compared with healthy controls, significant cortical thickness reductions were observed in multiple brain regions in mental retardation patients. These regions included the bilateral lingual gyrus, the bilateral fusiform gyrus, the bilateral parahippocampal gyrus, the bilateral temporal pole, the left inferior temporal gyrus, the right lateral orbitofrontal cortex and the right precentral gyrus.

Our finding of reduced cortical thickness in mental retardation is consistent with previous studies. For instance, Moorhead et al. reported reduced gray matter volume in multiple brain regions in patients with mental retardation, including the parahippocampal gyrus, the fusiform gyrus, inferior temporal gyrus and precentral gyrus [12]. Lower regional gray matter volumes have also been reported in the parahippocampal gyrus and precental gyrus in patients with fragile X syndrome, compared with typically developing boys [19]. In another study on human intelligence, Narr et al. reported positive correlations between IQ and cortical thickness in bilateral prefrontal, bilateral temporal cortices (inferior temporal, fusiform, and parahippocampal cortices) and right extrastriate occipital (lingual gyrus) cortical regions in healthy subjects[14], which, to some extent, support our finding. In addition, these regions have been implicated in the functioning of working memory. Briefly, the parahippocampal gyrus and the lingual gyrus have been reported to be involved in spatial information processing as well as novelty and memory encoding [20], [21], [22]. Activations in the parahippocampal gyrus, the lingual gyrus, the fusiform gyrus and the inferior temporal gyrus have been documented in many working memory studies [23], [24], [25]. Therefore, the observed cortical thickness reductions in these regions might be associated with the deficits in working memory, which were frequently reported in mental retardation [26], [27], [28], [29].

Cortical thickness reductions correspond to substantial pathological changes. Measuring the cortical thickness may provide important information about the integrity of the cerebral cortex. In mental retardation, cortical thickness reductions could result from various mechanisms. Firstly, the cortical thickness reductions might be due to primary developmental histopathological abnormalities, including defective neuronal generation or migration, cell density, and microcolumnar changes [30]. Classically, the central nervous system development is divided into three major stages: neuronal generation, migration, and differentiation/maturation[31]. Aberrations in one or more of these stages can have long-term consequences for the integrity of higher cognitive abilities [31], [32]. Many previous studies have reported developmental abnormalities in patients with mental retardation. In a review, Schaefer et al. described the common brain malformations associated with developmental abnormalities in patients with mental retardation[30]. In patients with unclassified mental retardation, many studies reported reductions in number and length of dendritic branches as well as aberrant morphology and number of dendritic spines[7], [8]. Additionally, abnormal dendritic spine characteristics have also been observed in both fragile X knockout mice and patients with fragile X syndrome [6], [33], [34]. Alternatively, the observed cortical thickness reductions in patients with mental retardation could be a secondary consequence of a lack of input to specific brain areas resulting from primary white matter abnormalities[35]. Using various neuroimaging techniques, white matter abnormalities have been intensively investigated in patients with mental retardation. For instance, many MRI studies have reported anomalies of corpus callosum and alterations of the white matter in patients with mental retardation [3], [4]. Diffusion tensor imaging studies also showed that patients with mental retardation have significantly lower fractional anisotropy (FA) than healthy controls in many white matter fiber tracts[36], [37]. Therefore, focal damage to the white matter and its connectivities in mental retardation patients could be responsible for the cortical thickness reductions in appropriate regions of the cerebral cortex.

For decades, it has been known that mental retardation may result from both harmful environmental and genetic factors during the developmental process [38]. Both of these factors may have an effect on the cortical thickness. Although some environmental factors that are often involved in mental retardation have been excluded in this study, it is still possible that other harmful environmental factors might have an effect on the cortical thickness [31], [32], [39]. On the other hand, genetic factors may play a critical role in abnormal cortical development. In fact, mutations in some genes implicated in mental retardation, such as fragile X mental retardation 1 gene, oligophrenin 1, p21-activated kinase, and rho guanine nucleotide exchange factor 6, have been found to directly and indirectly affect laminar organization, axonal guidance, and proper connectivity [1], [38], [40], which, in turn, might have an effect on the cortical thickness in patients with mental retardation. From this cross-sectional observational study, it is not possible to determine which factor plays a leading role in causing the cortical thickness reductions. Future studies will need to discriminate the role of specific genetic and/or environmental factors in the development of the cortical thickness reductions in mental retardation by using samples with higher homogeneity or animal experiments.

In conclusion, we found significantly reduced cortical thickness in multiple brain regions in patients with mental retardation compared with healthy controls. The observed cortical thickness reductions in these regions might be the anatomical substrates for the sub-average functioning and deficits in adaptive behaviors in mental retardation.

## Materials and Methods

### Subjects

All subjects of this study were chosen from subjects who participated in a study by Yu et al.[36]. The fifteen patients with idiopathic mental retardation are described in detail by Yu et al. [36] and two of them were discarded due to segmentation errors. In brief, the mental retardation patients were recruited from Beijing Huiling community service for people with disabilities and Beijing Lizhi recovery center for people with disabilities. All patients were diagnosed by an experienced psychiatrist according to the Diagnostic and Statistical Manual of Mental Disorders criteria for mental retardation: (1) IQ of approximately 70 or below on an individually administered IQ test; (2) at least two affected areas: communication, self-care, home living, social/ interpersonal skills, use of community resources, self-direction, functional academic skills, work, leisure, health and safety; (3) onset prior to age 18 years. Exclusion criteria included prenatal events (such as congenital infections, prolonged maternal fever in the first trimester, exposure to anticonvulsants or alcohol, and untreated maternal phenylketonuria), notable dysmorphology, near-drowning, traumatic brain injury, phenylketonuria, hypothyroidism and disorders known to be associated with mental retardation, such as neurofibromatosis and tuberous sclerosis. Patients with visible brain lesions on conventional magnetic resonance images were also excluded from this study. For comparison with the 13 mental retardation patients, 26 age- and gender-matched healthy subjects were included. FSIQ score was measured by means of the Chinese Revised Wechsler Adult Intelligence Scale (Table 2).

**Table 2:** Demographics of the samples.

	Normal controls (n = 26)	Mental retardation patients (n = 13)
Age (years)	23.4(4.6)*	22.6(2.3)*
Sex (male/female)	16/10*	8/5*
FSIQ	108.1(8.6)	50.0(10.0)

Data are mean (SD).

*no significant difference between groups (p>0.05).

### Ethics Statement

After a full explanation, parents of the mental retardation patients and all of the healthy controls gave voluntary and informed consent in written form according to the standards set by the ethical committee of Xuanwu Hospital of Capital Medical University, who specifically approved this study.

### MRI Data Acquisition

Three dimensional structural MRI scans were obtained on a 3.0 Tesla magnetic resonance scanner (Trio system; Siemens Magnetom scanner, Erlangen, Germany) with magnetization prepared rapid acquisition gradient echo. Detailed scan parameters were as follows: repetition time  = 2000 ms, echo time  = 2.6 ms, slice thickness  = 1mm, no gaps, flip angle  = 9°, matrix  = 256×224, field of view  = 256×224 mm^2^, 1×1 mm^2^ in-plane resolution.

### Preprocessing

Each scan was processed using FreeSurfer (http://surfer.nmr.mgh.harvard.edu/ ) using the volume and surface pipeline [41], [42]. Starting from the segmentation of the white matter and the tessellation of the grey/white matter boundary, an initial surface was obtained after automated topological correction. This surface was used as the initial shape for the deformable model that was used to reconstruct the pial surface. When all of the surfaces had been reconstructed, the cortical thickness was computed. The thickness was measured in the native space of each subject. The thickness was defined at each point on the pial surface (as well as its counterpart on the grey/white matter surface because of the one-to-one correspondence) as the mean of the two shortest distances [43] ; one was from the point on the pial surface to the grey/white surface, and the other was from the point on the grey/white matter surface to the pial surface. To compare cortical thicknesses point by point and to visualize the statistical results, the establishment of point correspondence across subjects in a standard surface-based coordinate system was required. Surface-based registration was used to build an average template, and all of the individual reconstructed cortical surfaces were aligned to this template [44]. Then, the cortical thickness data were resampled for each subject. Prior to statistical analysis, a heat kernel with a 30-mm width was used to smooth the cortical thickness maps to increase the signal-to-noise ratio and to improve the ability to detect morphometric variations [45].

### Statistical Analyses

Vertex-by-vertex contrasts of cortical thickness were performed for normal controls vs patients with mental retardation using SurfStat package (http://www.math.mcgill.ca/keith/surfstat/). Specifically, each contrast was entered into a vertex-by-vertex GLM including diagnosis, sex, and exact age as covariates. Subsequently, a corrected vertex-wise P value was obtained using random filed theory [46]. The level of significance for vertices was set at a conservative, surface-wide P<0.05 after multiple comparison correction. Only clusters with a minimum of 100 vertices were reported.
